# Radiation Pneumonitis: Old Problem, New Tricks

**DOI:** 10.3390/cancers10070222

**Published:** 2018-07-03

**Authors:** Varsha Jain, Abigail T. Berman

**Affiliations:** Department of Radiation Oncology, University of Pennsylvania, Philadelphia, PA 19104, USA; Abigail.Berman@uphs.upenn.edu

**Keywords:** radiation pneumonitis, radiation therapy, lung cancer

## Abstract

Radiation therapy is a major treatment modality for management of non-small cell lung cancer. Radiation pneumonitis is a dose limiting toxicity of radiotherapy, affecting its therapeutic ratio. This review presents patient and treatment related factors associated with the development of radiation pneumonitis. Research focusing on reducing the incidence of radiation pneumonitis by using information about lung ventilation, imaging-based biomarkers as well as normal tissue complication models is discussed. Recent advances in our understanding of molecular mechanisms underlying lung injury has led to the development of several targeted interventions, which are also explored in this review.

## 1. Introduction

Radiation therapy (RT) is a major treatment modality for management of non-small cell lung cancer (NSCLC). The lung is a radiosensitive organ and the effectiveness of delivering radiation to tumors arising within has been largely limited by normal tissue injury. Radiation pneumonitis (RP) is one of the most clinically challenging toxicities following lung radiation. The time to onset and the severity of RP is dependent on a number of patient and treatment related factors. In this review, we discuss some of these factors.

We focus on emerging data that can aide in patient selection, treatment planning and management. Recent advances in functional imaging have led to the exploration of imaging-based biomarkers that can help in identifying patients at increased risk of RP. These technologies can also be used to get information about lung ventilation to limit dose to the well-ventilated lung with the goal of minimizing toxicity. We also discuss the use of normal tissue complication probability (NTCP) models for treatment planning and selection of treatment techniques. Classically, glucocorticoids have been the cornerstone of clinical management of RP. However, advances in our understanding of molecular mechanisms underlying lung injury has led to the development of several promising pharmaceutical interventions, which are also discussed in this review.

## 2. Pathogenesis and Clinical Manifestations

Radiation induced pulmonary effects can be varied and often long-lasting. These include edema, epithelial degeneration and subsequent regeneration, invasion of alveoli by bronchial epithelium, endothelial sloughing, disruption of microvasculature, and atelectasis [1]. Some of these direct and indirect effects can begin within nanoseconds of radiation exposure through induction of free radicals and the accompanying oxidative stress [2]. In certain instances, this injury fails to fully repair/resolve. The tissue enters a progressive and dysregulated process that can manifest as acute and late endpoints of RP and fibrosis [3,4,5].

Clinically, RP can manifest within weeks to months following radiotherapy. However, most cases are reported within the first 8 months. The classic triad of symptoms includes dyspnea on exertion, non-productive cough, and hypoxemia; though low-grade fevers are also common. Physical exam findings can include pleural friction rub, moist rales as well as signs of consolidation [6]. These manifestations may be complicated by the presence of pre-existing lung disease such as COPD or emphysema. The incidence of symptomatic RP is estimated to be in the range of 15–40% [7]. The risk of mortality associated with RP is however low, and is estimated to be <2% [8]. 

Objectively, the effect of RP on lung function can be assessed using pulmonary function tests (PFTs), a clinical gold standard. Borst et al. [9] reported a decrease in first expiratory volume in 1 s (FEV1) as early as three months post RT with continued deficits observed at 18 and 36 months. The decrease in PFTs was dependent on mean lung dose (MLD) and underlying pulmonary conditions such as COPD. Diffusion capacity of lungs (DLCO) is another important PFT parameter affected by RP. Variations in this parameter are also dose dependent with effects seen for MLD as low as 13 Gy [10]. In a prospective study of 128 patients with good long term follow up, DLCO decreased progressively at an annual rate of 3.5%/year [11]. Such impairments in DLCO have been reported by several other groups. However, these changes in PFTs are dependent on tumor location and size. For example, in one observational study of 82 patients with locally advanced lung cancer, improvement in both FEV1 and DLCO were noted 3–4 months after radiation. Multivariate analysis revealed that reduction in tumor size was correlated with these improvements [12]. 

## 3. Grading

Several grading systems for evaluation of RP have been utilized (Table 1). These grading systems factor in a combination of clinical, functional and radiographic changes that may occur following lung radiation. The most commonly used grading system is the Common Terminology Criteria for Adverse Events (CTCAE) version 4.0 published by the National Cancer Institute [13]. Other scoring systems are available from the Radiation Therapy Oncology Group (RTOG), European Organization for Research and Treatment of Cancer (EORTC) [14], Southwest Oncology Group (SWOG) [15], Eastern Cooperative Oncology Group [16] and World Health Organization [17]. In most of these systems, RP is graded from 1 to 5. Grade 1 typically represents mild symptoms not requiring medical intervention. Grade 4 and 5 represent lethal/life-threatening toxicity including death. There is some heterogeneity in the definition of Grade 2 and 3 toxicities; though typically Grade 2 represents needing some medical intervention (for example steroids) and Grade 3 indicates the use of supplemental oxygen.

## 4. Factors Associated with Radiation Pneumonitis

### 4.1. Patient Related Factors

Several patient related factors such as age, gender, performance status, smoking status and underlying pulmonary conditions have been implicated in increasing chances of developing RP. Much of the data, however, is based on retrospective single institution series, with a heterogeneous study population and insufficient statistical power to detect differences. A meta-analysis published by Vogelius et al. studied some of these clinical factors in a systematic fashion [18]. They synthesized the data from 31 independent studies with available odds ratio (OR) data for RP. Their results demonstrated that advanced age (OR 1.66, *p* < 0.0001) and pulmonary comorbidities (OR 2.27, *p* = 0.007) were significantly associated with the risk of developing RP. They also found that ongoing smoking protects against RP (*p* = 0.008) and a history of smoking shows borderline to significant protection (*p* = 0.006). Of note, most of the included studies used Grade 1 or lower RP as their primary endpoint. Several possible explanations have been proposed for this association between smoking status and RP. These include decreased inflammatory reaction among smokers [19], smoking associated hypoxia [20], resistance against oxidative stress and decreased capacity to repair DNA damage in non -smoking patients [21]. Also, smokers are more likely to have pulmonary symptoms at baseline, and hence less likely to recognize and report symptoms.

Pre-existing lung conditions are common in patients undergoing thoracic radiation for non- small lung cancer and an important risk factor for development of RP. Lung pathologies are broadly classified as obstructive or restrictive in nature. Asthma and chronic obstructive pulmonary disease (COPD) fall in the former category while emphysema and interstitial lung disease (ILD) comprise restrictive lung diseases. The literature on the effect of COPD on RP is mixed, with some studies even reporting it to be a negative predictive factor for RP [22]. However, ILD has been universally associated with increased risk of RP and in some cases associated with fatal RP.

Clinically, extensive ILD is sometimes considered a contraindication for thoracic RT. Bahig et al. [23] published one of the largest retrospective series of thoracic stereotactic body radiation (SBRT) and ILD. They evaluated 504 patients with SBRT, 28 of whom had ILD. Rate of Grade 3 or higher RP was 32% in the ILD group vs. 2% for patients without ILD (*p* < 0.001). There were five reported instances of RP, all of which occurred in the ILD group. In another large retrospective series of 651 patients treated with conventional thoracic RT, 78% of patients who developed RP had pre-existing ILD [24].

Recent reports have implicated even subclinical ILD to be associated with severe RP. Subclinical ILD refers to having radiographic features of ILD without symptoms or a clinical diagnosis of ILD. In a series of 100 patients who underwent SBRT, subclinical ILD was detected in 16 patients, of whom 19% had extensive RP (*p* = 0.004). One patient suffered fatal RP and had both subclinical ILD and emphysema [25]. In a series of 62 patients undergoing conventional thoracic radiation, Yamaguchi et al. identified 11 patients with subclinical ILD, of whom 36% developed symptomatic RP (*p* = 0.027) Two of these patients suffered fatal RP [26].

Patients with clinical/subclinical ILD have a greater risk for RP even when standard dosimetric lung parameters (such as mean lung dose and V20) are met. Patients should be counseled about the additional risk of RP and every effort should be made to reduce the lung dosimetric parameters to as low a value as reasonably achievable.

### 4.2. Treatment Related Factors

General management of lung cancers typically involves a combination of surgery, chemotherapy and radiation. While early stage disease can be treated definitively with surgery or radiotherapy, a combined multimodality approach remains the mainstay of treatment for locally advanced disease.

#### 4.2.1. Chemotherapy

Chemotherapy can have a synergistic effect with radiotherapy and is commonly used in management of locally advanced NSCLC (LA-NSCLC). However, the influence of sequencing of chemotherapy and radiotherapy on RP has yielded mixed results. Concurrent treatment is the current standard of care as it is associated with better disease related outcomes and an overall survival benefit [27]. However, sequential chemotherapy and radiation is sometimes considered in situations such as large treatment volumes (hence inability to meet standard dosimetric constraints upfront), poor performance status patients or in post-operative situations. The rationale for this is the thought that a more protracted treatment course would minimize treatment associated toxicity. However, in a 2012 meta-analysis, Vogelius et al. showed that sequential chemoradiation (CRT) was associated with a higher risk of RP than concurrent CRT (OR 1.6, *p* = 0.01) [18]. While the reason for this observation is unclear, it is possible that patients undergoing concurrent radiation were more likely to get lower doses of RT due to concern for higher toxicity. Additionally, the data may be biased as sequential schedules are typically preferred in patients with poor performance status, elderly and those with comorbidities. These patients groups have a higher baseline risk of developing RP. Furthermore, the biological effects of RT can continue for several weeks after the last exposure and it is possible that the interaction between chemotherapy and radiation does not disappear even by separating the modalities in time.

Several studies have also looked into the risk of RP based on the choice of chemotherapy [28,29]. A recent meta-analysis by Palma et al. reviewed more than 70 internationally published articles to develop and validate a predictive model for RP in patients receiving modern concurrent CRT for LA-NSCLC [8]. On recursive partitioning analysis, the highest risk of RP (> 50%) was seen in patients >65 years of age and receiving carboplatin/paclitaxel.

#### 4.2.2. Immunotherapy

The introduction of immunotherapy has revolutionized the management of NSCLC. These agents have been demonstrated to be superior to standard cytotoxic chemotherapy in all comers with advanced disease in a second line setting [30,31,32]. Furthermore in patients with high levels of programmed death ligand (PD-L1) expression, these agents might have a role in upfront/first line setting as well [33]. Recently, darvalumab, a selective human IgG1 monoclonal antibody that blocks PD-L1 binding to programmed death (PD-1), was approved by the Food and Drug Administration (FDA) for consolidative therapy after definitive CRT. This approval was based on the favorable results of the PACIFIC trial (discussed below) [34]. The coming decades are bound to see an increasing use of immunotherapy for management of NSCLC and understanding its toxicities both alone and in conjunction with RT is imperative.

Immunotherapy is independently associated with an increased risk of pneumonitis. In a recently published large retrospective study of over 1800 patients, immunotherapy was associated with the development of ILD in 3.5% patients [35]. In the same vein, a recent meta-analysis reported an overall incidence of 2.7% and 0.8% for grade 3 or higher pneumonitis, respectively, in patients treated with immunotherapy [36]. This incidence is higher than what has been observed with other drugs used to treat NSCLC, for example, premetrexed, erlotinib, gefitinib, docetaxel, gemcitabine or crizotinib [37,38,39,40,41,42]. However, it remains uncertain if this risk is synergistic with RT. RT has been demonstrated to prime the immune response and hence can potentiate the effects of immune therapy [43].

There is currently sparse data (mostly case reports) on the risk of pneumonitis in patients receiving concurrent immunotherapy and RT. However, some recent trials have elucidated the risks with a sequential approach. A phase 1 trial, KEYNOTE-001, studied the use of pembrolizumab, a monoclonal antibody that blocks the interaction between PD-1 and its ligands, in patients with advanced and metastatic NSCLC. A secondary analysis of this study demonstrated that the incidence of pulmonary toxicities of any grade was higher in patients who had received previous RT versus those who did not (63% vs. 40%, *p* = 0.052). However, there was no difference in Grade 3 or higher pulmonary toxicity [44]. In the previously mentioned PACIFIC study, patients were randomized to receiving durvalumab or placebo in a 2:1 ratio following definitive CRT. While the risk of pneumonitis of any grade was higher with durvalumab (33.9% vs. 24.8%), the incidence of clinically important Grade 3 or 4 events was well balanced between the groups (3.4% in the durvalumab group vs. 2.6% in the placebo group) [34].

#### 4.2.3. Surgery

RT can be delivered in both adjuvant as well as neoadjuvant settings for management of NSCLC. Radiotherapy is given adjuvantly after surgery (post-operative radiotherapy, PORT) in certain situations where the risk of regional recurrence is high (e.g., positive margins, mediastinal node positive disease). Kocack et al. prospectively looked at 147 patients who received radiotherapy for management of lung cancer with a 6 month follow up [45]. About 30% of these patients received surgical intervention before radiotherapy which included pneumonectomy, lobectomy, wedge resection and exploration without resection. Interestingly, they found no statistically significant difference in the rate of RP based on the extent of resection. This was also confirmed in a meta-analysis which included five additional studies which looked at effect of type on surgery on RP [18].

However, contrary to the above findings the type of surgery was shown to effect outcomes in a study exploring the benefit of neoadjuvant CRT followed by surgery [46]. They observed that overall survival was improved in the cohort receiving tri-modality therapy with CRT followed by a lobectomy compared to patients receiving CRT alone. They also reported on the general pulmonary toxicity and reported that pneumonitis/Grade3 or 4 respiratory complications were seen in 9% of patients in the lobectomy arm compared to 14% in the CRT alone arm. This indicated that if a less radical operation such as a lobectomy (instead of a pneumonectomy) can be performed, it is less likely to cause RP than a course of definitive CRT.

#### 4.2.4. Radiation Therapy

The dose and the volume of lung irradiated are important parameters in the possible pathogenesis of RP. In this section we will discuss the dosimetric correlates associated with higher risk of RP as well as the choice of treatment technique and modality.

##### Dosimetric Correlates

There is a wealth of literature reporting correlations between radiation dosimetric parameters and RP. Most studies have validated V20 and mean lung dose (MLD) as the most frequently correlated parameters, though several other variables have also shown to be predictive, including volume of lung receiving ≥ 5 Gy (V5), ≥ 13 Gy (V13), ≥ 25 Gy (V25) and ≥ 30 Gy (V30) [8]. However, studies have shown that these parameters tend to be very collinear (that is, an increase in one parameter, for example V20, will lead to an increase in other parameters) and differences in predictive value among different dosimetric variables tends to be small.

There is accruing data (mostly from animal models) that rather than the pulmonary dose alone, the combined dose to the heart and lungs might have a synergistic effect on the risk of developing RP [47,48]. Huang et al. developed a model for predicting the risk of RP in 209 patients with LA-NSCLC) and found that heart dosimetric variables were important in addition to lung variables [49].

A recently published study using a bootstrap modeling approach, however, it failed to confirm the above findings. The authors concluded that the incidental dose to the cardiac atria and the ventricles did not seem to improve RP risk prediction in their cohort of 188 patients with LA-NSCLC treated with intensity modulated radiotherapy (IMRT) [50].

##### Treatment Technique: 3D vs. IMRT

IMRT has a number of theoretical advantages over 3D-RT for management of LA-NSCLC. It provides better dose conformality, and hence can improve radiation coverage of tumors and enhance the therapeutic ratio by avoiding adjacent organs at risk (OARs). Several retrospective dosimetric studies have been published on the benefit of IMRT [51,52]. Recently a prospective Phase I study of patients undergoing definitive IMRT for treatment of LA-NSCLC with corresponding 3D plans was published [53]. IMRT was associated with a global decrease in normal tissue exposure. There was also a statistically and clinically meaningful reduction in V20 Gy (21.5% vs. 26.5%, *p* < 0.01) and MLD (11.9 Gy vs. 14.9 Gy, *p* < 0.01), both of which are important metrics for predicting RP. Interestingly, this was achieved without compromising lung V5 Gy (*p* = 0.76).

While the above studies demonstrated the dosimetric advantage of IMRT, the highest-level data for the clinical benefit of IMRT comes from the secondary analysis of RTOG 0617—a dose escalation study. Chun et al. performed a secondary analysis of the above trial in which patients received concurrent CRT with or without cetuximab to a dose of 60 vs. 74 Gy [54]. In this trial, the choice of RT technique was at physician’s discretion with about 47% of the patients receiving IMRT. Because the treatment technique was not randomized, the IMRT group had larger and more advanced tumors. Despite this, patients in the IMRT group had significantly lower occurrence of Grade 3 or higher RP (7.9% vs. 3.5%, *p* = 0.039). The protective effect of IMRT for RP persisted in multivariate analysis (hazard ratio [HR], 0.44; *p* = 0.0653) and was particularly pronounced in large tumors that were bigger than the median size of 460 mL (HR, 0.22; *p* = 0.02). This data strongly supports the routine use of IMRT for management of LA-NSCLC.

##### Treatment Modality: Protons vs. Photons

**Figure 1:**
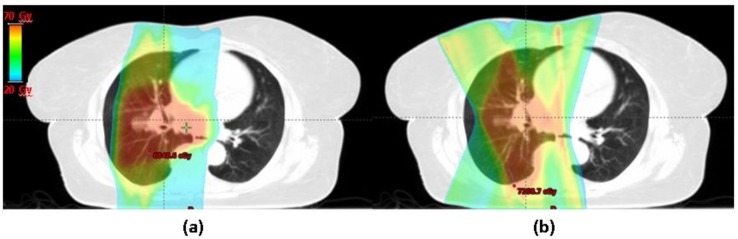
Representative images of a double scatter proton (**a**) vs. Intensity modulated radiation therapy IMRT photon (**b**) plans for a patient with locally advanced lung cancer. Note the differences in dosimetry between the two plans.

The benefit of protons in the management of NSCLC has been a topic of much controversy and debate for several years. Several retrospective series as well as virtual/in silico clinical studies have hypothesized the potential benefit of protons in terms of reducing the dose to normal tissue particularly the lung and heart and decreasing the likelihood of RP [55,56]. Representative images of a proton vs. photon plan for the treatment of locally advanced NSCLC are shown in Figure 1.

In a recently published National Cancer Database (NCDB) analysis, proton therapy was associated with a better five years overall survival as compared to photons on recursive partitioning analysis (RPA) analysis; 22% vs. 16% (*p* = 0.025), respectively [57]. The authors hypothesized that this was likely secondary to better tissue sparing achievable by protons; though acute and chronic toxicity data was not available for this analysis.

Earlier this year, Liao and colleagues published the first randomized trial to assess the value of proton therapy in management of LA-NSCLC [58]. The study was designed as a Bayesian trial which used an adaptive randomization using real time assessment of outcomes so that more patients were allocated to the better treatment arm if a difference was observed. The primary end point was the first occurrence of severe (Grade ≥3) RP or local failure (LF). The study failed to show superiority of protons in terms of Grade 3 or higher RP (10.5% with protons vs. 6.5% with photons) or local failure (10.5% with protons vs. 10.9% with photons). From a dosimetry perspective, there was no significant difference between typical parameters associated with RP, i.e., V20 and mean lung dose between patients treated with protons vs. photons. However, this advantage has been hypothesized in several retrospective and virtual studies mentioned prior.

Of note, lung V5 and mean heart dose was lower with protons and some argue that the latter is more likely to contribute to an overall survival benefit. This was not studied in the aforementioned trial. Additionally, the study required patients to meet dosimetric constraints for both proton and photon plans, which may have resulted in decreased enrollment of patients who would have most likely benefited from proton therapy. We await the results of RTOG 1308, a Phase III randomized trial comparing overall survival after proton versus photon CRT for inoperable LA-NSCLC; specifically studying RP as one of the secondary end points.

## 5. New Frontiers

Several recent advances in treatment planning technology, for example, use of more gantry angles, better imaging guidance and more accurate dose composition for moving targets, has helped to decrease dose to OARs. Furthermore, using information from 4D CT scans to reduce dose to the functional well-ventilated lung as well as using imaging biomarkers and NTCP models for RP risk assessment, can help us to further reduce RT related pulmonary toxicity.

### 5.1. Using 4D CT for Functional Avoidance Planning

Typically used normal tissue tolerance limits for radiation planning assume homogenous underlying lung function. However, several studies have demonstrated that there is significant functional heterogeneity across the lung volume which may be exacerbated in the presence of underlying lung pathology [59,60]. Studies have demonstrated that as high as 70% of patients undergoing thoracic radiation exhibit spatially variant lung function [61,62]. This has generated an interest in using different functional imaging modalities to identify areas of high functioning lung with the intent to avoid them in radiation planning.

One such method is 4D CT ventilation imaging which uses phase resolved CT images to calculate pulmonary ventilation [63,64]. This is a particularly appealing option as 4D CT scans have become standard of care for evaluation of tumor motion in patients undergoing thoracic radiation. Hence, these 4D CT ventilation images can be calculated at no extra financial or radiation exposure cost to the patient.

Brennan et al. validated 4D CT ventilation derived metrics of lung function with the clinical gold standard-PFT data. They found good correlation between the two methods (on the order of 0.7) and noted that 4D CT ventilation metrics were able to delineate between normal vs. abnormal lung function [65]. In a recent study, Faught et al. used a NTCP model to estimate the reduction in toxicity for CT ventilation based functional avoidance planning [66]. They retrospectively analyzed 70 patients who underwent 4D CT planning based thoracic radiation and demonstrated reductions as high as 7.1% and 4.8% in Grade 2+ and Grade 3+ RP, respectively, across the cohort. In individual patients, maximum absolute reduction of up to 52% and 16% were seen for Grade 2+ and Grade 3+ RP, respectively.

This retrospective work on 4D CT ventilation imaging has provided the framework for design of clinical trials using 4D CT, ventilation imaging for functional avoidance radiotherapy [67], NCT02528942. To provide practical data for guidance on assessment of patient eligibility, planning strategies and methods of assessment for functional treatment plans, Waxweiler et al. recently published a complete virtual trial using retrospective data from 96 LA-NSCLC patients [68]. Their functional planning demonstrated than an average reduction of 2.8 Gy to the mean lung dose can be expected relative to standard thoracic plans. Their findings provide salient data for development of future clinical trials in this area.

### 5.2. Imaging Based Biomarkers: Pre-Treatment FDG

There has been a recent interest in using imaging-based biomarkers to predict the risk of RP. Several groups have investigated using pre-treatment [18F]-2-fluoro-2-deoxyglucose positron emission tomography (FDG PET) imaging towards this end. The rationale for this is the thought that pretreatment inflammation in the lung would make pulmonary tissue more susceptible to radiation induced lung injury and hence RP. Pulmonary inflammation manifests as enhanced FDG uptake, hence allowing for quantitative assessment of RP.

One of the first investigations exploring this was a study by Petit et al. [69]. They performed a retrospective study of 101 patients with NSCLC and evaluated the correlation between symptomatic RP and pre-RT FDG PET evidence of pulmonary inflammation. They demonstrated that the 95th percentile of the standard uptake value (SUV_95_) within the lungs was predictive of RP on multivariate analysis (*p* = 0.016), suggesting that SUV_95_ can be used to predict the risk of RP during thoracic RT treatment planning. Several other groups have since corroborated their findings evaluating patients treated with various treatment modalities, including protons [70] and techniques such as SBRT [71].

Evaluation of pre-RT FDG uptake can be conveniently incorporated in our current treatment planning algorithm, as these scans are routinely obtained for staging of NSCLC and for target delineation for radiotherapy treatment planning. Hence SUV_95_, computed from imaging studies obtained for staging and treatment planning can be used to stratify toxicity risk without posing additional cost to the patients. This information can also serve as an aid in designing clinical trials studying radiation induced pulmonary toxicity, as an end point to ensure well balanced groups and exclude patients who are at very high risk of toxicity.

One of the limitations of this approach is the moderate specificity of PET; estimated to be on the order of 51% [70]. Thus, sometimes it might be hard to distinguish underlying lung inflammation from acute pathologies such as infection, trauma, etc. A way to improve the specificity of this method is to incorporate the information obtained from FDG PET scans with additional imaging features. In a recent publication, Anthony et al. incorporated CT lung texture features with SUV information to improve the classifier performance of a radiomics based model of RP [72].

### 5.3. NTCP Modeling for Risk Assessment

NTCP estimates the risk/probability of a given side effect as a function of increasing dose to an organ at risk (OAR) or increasing volume with an OAR receiving a certain dose. One such widely used models is the Quantitative Analysis of Normal Tissue Effects in the Clinic (QUANTEC) initiative, which performed a collective analysis on a number of published studies [73]. The authors formulated a relationship between the mean dose to the lungs (MLD) and the risk of RP for NSCLC patients and provided a quantitative estimate of the dose-response relationship.

A modification to the above model was proposed by Applet et al. [74]. In addition to the dosimetric factors, they incorporated clinical risk factors obtained from a large, published meta-analysis [18]. This helped to improve the predictive power of the model compared to a dose-only approach and was validated in a clinical cohort. Another commonly applied NTCP model is the Lyman model, which uses a binary (yes/no) toxicity evaluation endpoint [75]. Tucker et al. added single nucleotide polymorphisms to the standard Lyman model to enhance its predictive value for RP [76].

This model-based approach to prediction of toxicities can be useful in deciding which patients stand to benefit from a certain treatment modality. This is particularly relevant when there is no Level I data or randomized control trials supporting the use of a modality. For example, in the Netherlands this model based approach has been approved by the Health Care Insurance Board and is used to decide which patients will benefit from proton therapy [77]. Hence, protons are offered only to those patients who are most likely to profit from this modality.

## 6. Management of RP: Targets and Therapies

The mainstay of in clinic management of RP is with glucocorticoids; typically a dose of 60–100 mg/day of prednisone for 2 weeks followed by a slow taper over 3–12 weeks [6]. Supportive care with anti-tussive therapy, supplemental oxygen and antibiotics is also helpful. However, recent advances in our knowledge of molecular mechanisms underlying RP has led to identification of several potential targets for therapeutic intervention. These therapeutic targets have yielded promising data in animal models and some early phase clinical data. However, there is no Level I evidence supporting the routine use of these therapies in humans, though several investigations are underway.

### 6.1. Therapies Targeting Free Radical Production

Induction of free radicals is a classically recognized mechanism for radiation’s genotoxic effects. Several agents have been explored to reduce such radicals in normal versus tumor tissue and the only agent that is currently FDA-approved as a clinical radiation protector is amifostine. It is a thiophosphate whose active metabolite acts as a radical scavenger. It was initially clinically approved as a cytoprotectant for head and neck radiotherapy. However, it has proved less efficacious in other sites, with studies demonstrating both positive [78] and negative [79] effectiveness in the lung.

Another approach to minimize the effect of free radicals is to utilize our body’s own natural enzyme defense system against oxidative damage. This is regulated by activation of catalases, such as glutathione peroxidase and the superoxide dismutase (SOD) which convert the superoxide radical into oxygen and hydrogen peroxide prior to further metabolism [80]. Several groups have demonstrated anti-fibrotic properties of SODs in animal models [81,82]. In one the earliest reports of human application, researchers from Necker Hospital, Paris used a liposomal SOD (Lipsod) [83]. A regression in pulmonary fibrosis was noted in all of the 34 evaluated patients and the treatment was well tolerated.

Other naturally available anti-oxidant approaches have been explored as well. Dietary flaxseed and its bioactive lignan component have demonstrated radio-protective properties in a murine model; decreasing pulmonary fibrosis, inflammation, cytokine secretion and lung damage while enhancing mouse survival (40% vs. 70–88%) [84]. Given these promising results, an ongoing clinical trial at our institution is exploring the clinical benefit of dietary flaxseed in patients undergoing thoracic radiation (NCT02475330).

Another interesting plant based anti-oxidant approach that has provided some indication of success is the use of genistein. It is a soy isoflavone that acts both as a non-specific protein kinase inhibitor and also a ROS scavenger. Recent work has suggested that both single dose pre-treatment and chronic post radiation administration using genistein as part of a supplemented diet provides protection against the induction of RP, as well as a reduction in the extent of fibrosis [85,86].

### 6.2. Targeting Recruitment of Inflammatory Cells

Local inflammation is the predominant histological and physiological feature during the acute phase following radiation injury. This is mediated by macrophage infiltration into the air spaces and focal accumulations of mononuclear cells and mast cells. This accounts for the observed benefit of glucocorticoids which can limit this inflammatory reaction.

Studies have also investigated the use of cyclooxygenase-2 (COX-2) inhibitors such as Celecoxib, based on their anti-inflammatory potential and some preliminary reports of overexpression of COX-2 in lung cancer [87]. However, the results have been disappointing with benefit limited to a few patients only. Another category of drugs with initial promising results is statins (HMG-CoA-reductase). While their clinical role as a lipid lowering agent is well established, recent studies have demonstrated a significant anti-inflammatory function as well [88]. On a genetic level, statins have shown the ability to reverse radiation induced dysregulation of gene expression (such as p53) [89]. In a cohort of patients undergoing pelvic RT, Wedlake et al. demonstrated that statins were associated with decreased radiation induced acute GI symptoms and exhibited long term protective effects [90].

### 6.3. Targeting Cytokine and Growth Factor Expression

Radiation mediated tissue injury is known to induce changes in the expression levels of cytokines such as tumor necrosis factor (TNF-α), IL-1 and IL-6 as well as growth factors such as transforming growth factor (TGF-β) [91]. Specifically in the lung, animal radiation models have shown that IL-1 is elevated in the bronchoalveolar lavage (BAL) [92]. Clinically, Chen et al. demonstrated the feasibility of applying IL-1α and IL-6 measurements of blood specimens to predict RP [93].

TGF-β is a highly pleiotropic molecule with a role in regulation and inhibition of cell growth and immunosuppression. Early expression of TGF-β is seen during the immediate post radiation period and is thought to be a part of a homeostatic mechanism as the tissue attempts to regulate the wound-healing response [2]. Recent work has suggested an association between TGF-β single nucleotide polymorphisms and the risk of pneumonitis [94]. Additionally, studies in murine models using long term administration of TGF-β inhibitor (SM16) showed it to be effective in reducing the extent of radiation induced lung injury [95]. While clinical data is lacking, this is a class of agent that may be worth investigating.

### 6.4. Other Miscellaneous Targets

The role of the Renin-Angiotensin System (RAS) in mitigating lung injury was discovered rather serendipitously. RAS inhibitors have classically been used as anti-hypertensives given their role in short and long term regulation of arterial blood pressure. Several studies have indicated the potential role of angiotensin converting enzyme inhibitors (ACEi) in alleviating radiation induced lung injury [96,97]. In a clinical randomized controlled trial, the application of captopril, an ACEi was associated with a reduction in pulmonary related mortality resulting from total body irradiation [98]. Of note, captopril is a special type of ACEi as the sulfahydryl group in its molecular structure is capable for scavenging radicals, and thus can provide an additional mechanism for preventing lung injury [99]. Larger retrospective series in patients on ACEi has also yielded favorable results [100].

## 7. Conclusions

RP is a dose limiting toxicity of radiotherapy for management of NSCLC. The risk of development of RP as well as its severity is dependent on several patient and treatment related factors, including dosimetric factors and combining chemotherapy. A recently published Bayesian randomized trial aimed at determining the benefit of protons for reduction of pulmonary toxicity had negative results. New frontiers for minimizing the incidence of RP are being explored. Information about heterogeneity in lung ventilation can be obtained from 4D CT scans and used to minimize dose to the well-ventilated lung. Imaging-based biomarkers from PET scans can be used to identify patients at increased risk of pulmonary toxicity. NTCP models can also aid in risk stratification, patient selection and treatment planning. With advances in our understanding of molecular mechanisms underlying tissue injury, novel approaches for prevention and treatment of RP are on the horizon.

## Figures and Tables

**Table 1:** Demonstrates some of the commonly used grading scales.

Criteria	Grade 1	Grade 2	Grade 3	Grade 4	Grade 5
**CTCAE v4.0**	Asymptomatic	Symptomatic; Required medical intervention; Limits ADLs	Severe symptoms; Oxygen indicated; Impairs ADLs	Life threatening respiratory dysfunction	Death
**RTOG**	Mild symptoms	Persistent symptoms requiring symptomatic treatment	Severe symptoms, possibly requiring intermittent O_2_ or steroids	Severe symptoms requiring continuous O_2_ or assisted ventilation	-
**EORTC (LENT-SOMA)**	Asymptomatic or mild symptoms; slight imaging changes	Moderate symptoms; patchy imaging changes	Severe symptoms; increased density imaging changes	Severe symptoms requiring continuous O_2_ or assisted ventilation	Death
**SWOG**	Imaging changes; mild symptoms without steroids	Symptoms requiring steroids or tap for effusion	Symptoms requiring oxygen	Symptoms requiring assisted ventilation	Death

CTCAE 4.0, common terminology criteria for adverse events, version 4.0; RTOG, Radiation Therapy Oncology Group; EORTC, European Organization for Research and Treatment of Cancer; LENT-SOMA, late effects in normal tissue-subjective objective management analysis; SWOG, Southwest Oncology Group; ADL, activities of daily living.
